# Assessment of Intraseasonal Variation in Hospitalization Associated With Heat Exposure in Brazil

**DOI:** 10.1001/jamanetworkopen.2018.7901

**Published:** 2019-02-08

**Authors:** Qi Zhao, Shanshan Li, Micheline S. Z. S. Coelho, Paulo H. N. Saldiva, Kejia Hu, Michael J. Abramson, Rachel R. Huxley, Yuming Guo

**Affiliations:** 1 Department of Epidemiology and Preventive Medicine, School of Public Health and Preventive Medicine, Monash University, Melbourne, Australia; 2 Institute of Advanced Studies, University of São Paulo, São Paulo, Brazil; 3 Institute of Island and Coastal Ecosystems, Ocean College, Zhejiang University, Zhoushan, China; 4 College of Science, Health and Engineering, La Trobe University, Melbourne, Australia

## Abstract

**Importance:**

The onset of the hot season is known to be adversely associated with a range of health outcomes. However, little is known about whether the association is constant over the course of the hot season.

**Objective:**

To quantify the change in the association between heat exposure and hospitalization from the early to late hot season in the Brazilian population.

**Design, Setting, and Participants:**

This time-stratified case-crossover study used daily data on hospitalization and weather conditions during the 2000 to 2015 hot seasons in 1814 Brazilian cities. There were 49 145 997 admissions during the study period. Data analysis was conducted between May 12, 2018, and July 2, 2018.

**Exposures:**

Increase in daily mean temperature.

**Main Outcomes and Measures:**

Daily hospitalizations were recorded. Conditional quasi-Poisson regression with time-varying constrained distributed lag model was used to examine the city-specific association between heat and hospitalization in the early or late hot season. City-specific estimates were then pooled at the national level using random-effect meta-analysis. Stratified analyses were conducted by 5 regions, sex, 10 age groups, and 7 cause-specific categories.

**Results:**

Of the 49 145 997 admissions (59% women), the median (interquartile range) age was 33.3 (19.8-55.7) years. At the national level, the risk of hospitalization increased by 4.6% (95% CI, 4.3%-4.9%) and 2.3% (95% CI, 1.9%-2.6%) for every 5°C increase in daily mean temperature in the early and late hot season, respectively. Exposure to early heat was associated with greater risk of hospitalization for residents in the northeast (6.4%; 95% CI, 5.5%-7.3%) and central west (7.1%; 95% CI, 6.1%-8.2%) compared with other regions. Children aged 0 to 9 years and elderly individuals (aged ≥80 years) were most susceptible. Admissions due to endocrine, nutritional, and metabolic diseases were most strongly associated with heat exposure. There was an attenuation in the heat-associated risk of hospitalization from the early to late hot season for all subgroups except young children and patients with hospitalization caused by respiratory illness.

**Conclusions and Relevance:**

In this study, the association between heat exposure and hospitalization attenuated temporally for most of the Brazilian population. Preventive strategies to mitigate the association of high temperature with population health should focus in particular on the first few days of heat exposure.

## Key Points

**Question:**

During the hot season, is timing of heat exposure associated with varied risk of hospitalization in the Brazilian population?

**Findings:**

In this time-stratified case-crossover study, the association between heat exposure and hospitalization was greatest in the early hot season, particularly for residents living in the northeast and central west regions of Brazil. Exposure to early heat was associated with the highest risk of hospitalization for children and elderly individuals, and for admissions for endocrine, nutritional, and metabolic diseases.

**Meaning:**

Preventive strategies to mitigate the association of high temperature with population health should focus on extreme high temperatures occurring early in the hot season.

## Teaser

This time-stratified case-crossover study examines the association between timing of heat exposure and risk of hospitalization in the Brazilian population.

## Introduction

Globally, the past 30 years have constituted the hottest period since 1880, with this trend in global warming predicted to continue.^1,2^ Heat exposure during the hot season has been widely associated with excess morbidity and mortality from a range of health outcomes. For example, a study from the United States reported that every 2.8°C rise in daily mean temperature is associated with 9% excess hospitalizations for acute renal failure.^3^ Similarly, a study from the United Kingdom found a 2% to 4% increase in cardiovascular and genitourinary deaths per 1°C increase in daily mean summer temperature.^4^

Some previous studies exploring the association of heat exposure with health outcomes have assumed a constant susceptibility of the population to heat over the course of the hot season. However, a few recent findings have suggested that the association between heat and health may actually be greater at the beginning of the hot season than toward the end.^5,6,7^ If true, this could have implications for how hospitals and public health programs plan for the increase in demand for health services that is associated with high temperature, especially from population subgroups of highest heat susceptibility such as very young individuals, elderly individuals, and those with preexisting conditions.

Brazil is the fifth most populous country in the world and has experienced a greater-than-average increase in temperature over the past decades.^2,8^ In the past quarter century, Brazil’s rapid economic development has resulted in substantial increase in life expectancy and an associated increase in morbidity.^9,10^ We have previously shown that temperature anomalies are independent predictors for a variety of health-related conditions.^11,12^ In this study, we explore how the association between heat exposure and health outcomes changes according to timing of exposure. Specifically, using a nationwide hospitalization data set, we examine whether there is intraseasonal variation in the association between heat exposure and hospitalization in the Brazilian population.

## Methods

This study was approved and exempted by the Monash University Human Research Ethics Committee. The Brazilian Ministry of Health did not require ethical approval or informed consent for secondary analysis of aggregate anonymized data from the Brazilian Hospital Information System. This study followed the Strengthening the Reporting of Observational Studies in Epidemiology (STROBE) reporting guideline.^13^

### Data Collection

Between January 1, 2000, and December 31, 2015, daily data on hospitalization were collected through the Unified Health System from 1814 cities, which accounted for more than 78% of the national population. eFigure 1 in the Supplement shows the locations of 1814 cities in the 5 Brazilian regions, with the population coverage ranging from 26% in the north to 87% in the southeast. Variables included sex, age, date of hospitalization, and primary diagnosis (coded using *International Classification of Diseases, 10th Revision*). Daily hospitalization counts were then divided into men and women, 10 age groups (0-4, 5-9, 10-19, 20-29, 30-39, 40-49, 50-59, 60-69, 70-79, and ≥80 years), and 7 main cause-specific categories (eTable 1 in the Supplement).

Daily data on minimum and maximum temperatures were collected from a nationwide weather data set with a 0.25° × 0.25° resolution.^14^ Data from the center of each city were used in this study. Daily mean temperature was calculated by averaging daily minimum and maximum temperatures.^15^ Station-based data on relative humidity were collected from 193 cities during 2000 to 2012 via the Brazilian National Institute of Meteorology.

### Statistical Analysis

The hot season was defined as the hottest 4 consecutive months in each city. In this study, we explored the risk of hospitalization associated with heat exposure using daily mean temperature as it represents the average heat condition of a day. Associations between heat exposure and hospitalization in the early (first 2 months) and late (last 2 months) hot season were quantified using a 2-stage approach.

In the first stage, city-specific estimates of the association in the early or late hot season were quantified using a time-stratified case-crossover design. Briefly, we performed conditional quasi-Poisson regression with a time-varying constrained distributed lag model, with the time variable centered on the middle day of the first or last month of hot season.^5,16^ Stratum was defined as the same days of the week in the same calendar month for each case to control for temporal trend.^17,18^ Similar to previous studies,^6,19^ our initial analyses indicated that the association between heat exposure and hospitalization was linear and lasted for up to 7 days. We thus fitted the exposure-response association using a linear function for lag of 0 to 7 days and fitted the lag-response association using a natural cubic spline with 3 *df*. In this study, the confounding effect of public holidays was also controlled for using a binary variable.

In the second stage, city-specific estimates cumulated over a lag of 0 to 7 days were pooled at the national level using random-effects meta-analysis with maximum likelihood estimation. Residual heterogeneity from meta-analysis was quantified by the Cochran Q test and the *I*^2^ statistic. Stratified analyses were conducted by 5 regions, sex, 10 age groups, and 7 main cause-specific categories.

Sensitivity analyses were performed to examine the robustness of our results by changing the maximum lag of daily mean temperature from 7 to 9 days and the *df* of lag from 3 to 6. The confounding effect of relative humidity was also tested by adding the observed data in 193 cities. The linearity of the association between heat exposure and hospitalization was examined by using a distributed lag nonlinear model with a 3 *df* natural cubic spline for temperature dimension.

In this study, the estimated association between heat exposure and hospitalization was described as the percentage change in the risk of hospitalization (with 95% CI) for every 5°C increase in daily mean temperature. A random-effects metaregression model with the likelihood ratio test was applied to examine the differences in the association between heat exposure and hospitalization across region, population, and cause category subgroups in the early or late hot season, and the intraseasonal difference for each subgroup. For example, when examining the sex difference in the early hot season, a binary variable representing sex was set as the independent variable and city-specific estimates for men and women (in the early hot season) were set as the dependent variable. When examining the intraseasonal difference in the south, a binary variable representing early or late hot season was set as the independent variable and city-specific estimates (in the south) during the early and late hot season were set as the dependent variable.

Data analyses were performed using R statistical software version 3.4.1 (R Project for Statistical Computing). The package dlnm was applied to fit the cross-basis function in the first stage.^20^ The package mvmeta was applied to perform meta-analysis in the second stage and the metaregression.^21^ A 2-sided *P* value of less than .05 was regarded as statistically significant.

## Results

Between 2000 and 2015, the daily mean (SD) temperatures in the hot season were 25.3°C (2.1°C) at the national level, ranging from 23.4°C (1.3°C) in the south to 27.7°C (0.7°C) in the north. The daily mean temperature was slightly higher in the early hot season (Table). There were 49 145 997 hospitalizations in the 1814 cities during the study period, with women accounting for 59%. The median (interquartile range) age of patients was 33.3 (19.8-55.7) years. The rates of hospitalization were similar across 5 Brazilian regions, approximating to a mean of 2.1%.

**Table.** Summary of Hospitalizations and City-Specific Daily Mean Temperatures During the 2000 to 2015 Hot Seasons

Region	Population, No. (%)	Hospitalization Rate, %	Daily Temperature in the Hot Season, Mean (SD), °C		
			Whole	Early^a^	Late^a^
National	49 145 997 (100.0)	2.1	25.3 (2.1)	25.7 (2.6)	24.9 (3.0)
Region					
North	1 271 435 (2.6)	2.0	27.7 (0.7)	27.9 (1.7)	27.5 (1.3)
Northeast	13 823 251 (28.1)	2.1	27.1 (1.6)	27.4 (1.9)	26.8 (2.1)
Central west	3 847 427 (7.8)	2.2	26.3 (1.2)	26.6 (2.3)	26.1 (2.1)
Southeast	22 077 029 (44.9)	2.0	24.2 (1.3)	24.7 (2.)2)	23.7 (2.4)
South	8 126 855 (16.5)	2.2	23.4 (1.3)	24.0 (2.5)	22.8 (3.0)

^a^ The early hot season was the first 2 months of the hot season; the late hot season was the last 2 months of the hot season.

### Geographic Variation

**Figure 1.**
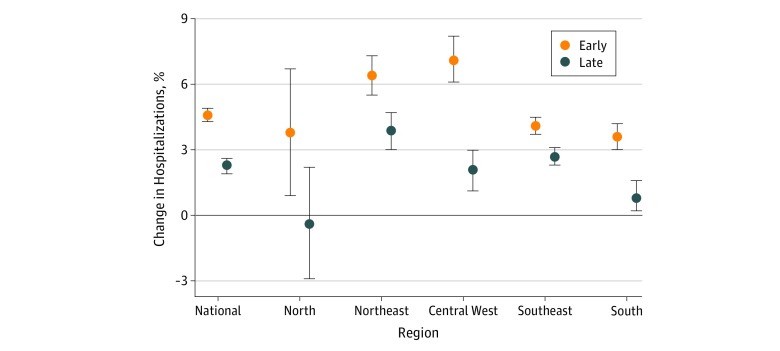
Association Between Heat Exposure and Hospitalization During the Early and Late Hot Season by Region Association was described as the percentage change in the risk of hospitalization for every 5°C increase in daily mean temperature. Error bars represent 95% confidence intervals.

For every 5°C increase in daily mean temperature in the early hot season, the estimated risk of hospitalization increased by 4.6% (95% CI, 4.3%-4.9%) at the national level (Figure 1). The risk of hospitalization associated with early heat was greatest in the central west (7.1%; 95% CI, 6.1%-8.2%) and northeast (6.4%; 95% CI, 5.5%-7.3%), and lowest in the south (3.6%; 95% CI, 3.0%-4.2%) (results of significant tests are shown in eTable 2 in the Supplement). The hospitalization risk increased by 2.3% (95% CI, 1.9%-2.6%) at the national level (but was nonsignificant in the north) for every 5°C increase in daily mean temperature in the late hot season. The strength of the association between heat exposure and hospitalization declined significantly within the hot season for all 5 Brazilian regions.

### Variation Across Demographic Subgroups

**Figure 2.**
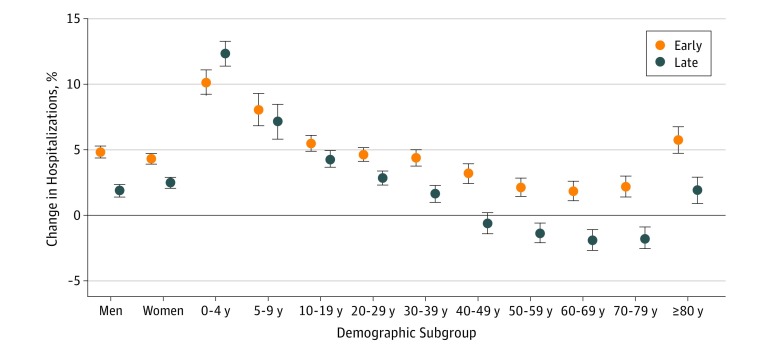
Association Between Heat Exposure and Hospitalization During the Early and Late Hot Season by Sex and Age Association was described as the percentage change in the risk of hospitalization for every 5°C increase in daily mean temperature. Error bars represent 95% confidence intervals.

The association between heat exposure and hospitalization was nonsignificantly greater for men (4.8%; 95% CI, 4.4%-5.3%) than for women (4.3%; 95% CI, 4.0%-4.7%) in the early hot season (Figure 2; eTable 3 in the Supplement). The opposite trend was observed in the late hot season between men and women. The risks of hospitalization associated with heat exposure during the hot season were greater for children aged 9 years or younger and for individuals aged 80 years or older than for middle-aged adults. The association declined across most population subgroups (being negative for those aged 50-79 years) in the late hot season. The exception was for children aged 0 to 4 years, whose heat-associated risk of hospitalization increased by 10.2% (95% CI, 9.2%-11.1%) and 12.3% (95% CI, 11.4%-13.3%) in the early and late hot season, respectively.

### Variation Across Cause-Specific Subgroups

**Figure 3.**
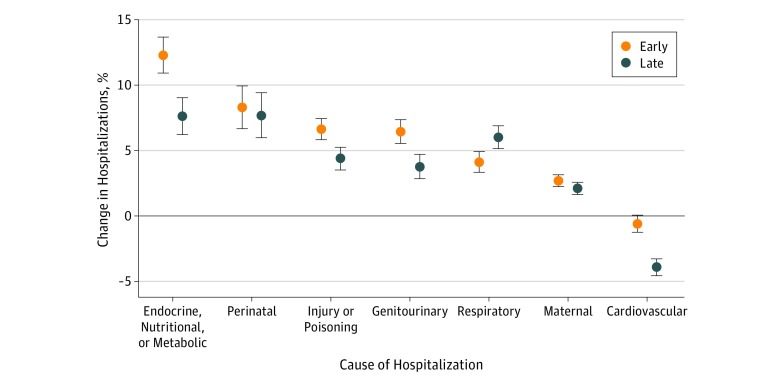
Association Between Heat Exposure and Hospitalization During the Early and Late Hot Season by Cause Category Association was described as the percentage change in the risk of hospitalization for every 5°C increase in daily mean temperature. Error bars represent 95% confidence intervals.

In the early hot season, the heat-associated risk was greatest for hospitalizations for endocrine, nutritional, and metabolic diseases and minimal for cardiovascular admissions (Figure 3; eTable 4 in the Supplement). The association between heat exposure and hospitalization attenuated significantly for most cause-specific categories (but inversely for cardiovascular diseases) in the late hot season. The exception was respiratory admissions, for which the risk associated with heat increased over the course of the hot season.

### Lag Patterns of the Association Between Heat and Hospitalization

For most geographic, demographic, and cause-specific subgroups, the risk of hospitalization associated with heat in the early hot season diminished within a lag of 0 to 2 days (eFigures 2-4 in the Supplement). In the late hot season and for most subgroups, the strength of the association between heat exposure and hospitalization within a lag of 0 to 2 days was similar to that in the early hot season, but the subsequent hospitalization deficits were more substantial. This phenomenon was particularly clear for individuals aged 50 to 79 years and for those admitted for cardiovascular causes. By contrast, the risk associated with heat within the first 2 days was greater in the late hot season for children aged 5 to 9 years and for respiratory admissions.

Results of sensitivity analyses indicated that our results were robust after changing the maximum lag from 7 to 9 days, changing the *df* from 3 to 6, and considering relative humidity (eTable 5 in the Supplement). The results of the distributed lag nonlinear model indicated a linear trend in the association between heat exposure and risk of hospitalization over the course of the hot season (eFigure 5 in the Supplement).

## Discussion

This is the first nationwide study, to our knowledge, to explore the change in the association between heat exposure and hospitalization over the course of the hot season by using a national data set comprising over three-quarters (>160 million) of the Brazilian population. In the early hot season, the association between heat exposure and hospitalization varied across Brazil, with a stronger association observed for residents in the central west and northeast compared with other regions. The risk of hospitalization associated with early heat was greatest for children and for elderly individuals. For the 7 main cause-specific categories, admissions due to endocrine, nutritional, and metabolic diseases were most susceptible to early heat exposure. The risk of hospitalization associated with heat lessened over the course of the hot season. The exceptions were for children aged 0 to 4 years and for respiratory admissions; in both instances, the risk of hospitalization associated with heat increased with increasing duration of the hot season.

Our main findings are broadly consistent with several surveys from populations in other continents, although with different exposure indicators, health outcomes, and methods.^5,22^ An ecological study evaluated mortality associated with heat exposure during the summers of 1985 to 2012 in 9 countries in North America, Western Europe, and East Asia by using a time-varying distributed lag nonlinear model for up to 10 days.^5^ The excess risk of mortality associated with extreme high daily mean temperature was higher in early summer, with the temperature associated with minimum mortality shifting upward temporally. Another European study observed a stronger intraseasonal reduction in heat-associated mortality in Mediterranean cities than in north-continental cities over a lag of 0 to 3 days during the summers of 1990 to 2000.^22^

The underlying mechanisms that mediate the association between timing of heat exposure during the hot season and hospitalizations are unclear and beyond the scope of this ecological study. It has previously been speculated that the intraseasonal attenuation may be due to physiological acclimatization whereby the body’s thermoregulatory response becomes more efficient with increasing duration of heat exposure.^23,24^ Some behavioral changes (eg, limiting time spent outdoors) by individuals in response to heat may explain some of the diminution in the association between heat exposure and hospitalization at the beginning of the hot season.^5,25^ Findings from the multicountry study may lend some support to this idea, as the risk of mortality associated with early heat was greater than the risk associated with late heat on a lag of 0 to 2 days, and this difference diminished with a longer lag period.^5^

In contrast, we observed opposite lag patterns for the association between heat exposure and hospitalization over the course of Brazil’s hot season. For most subgroups, the strengths of the association for early and late heat were similar on a lag of 0 to 2 days, but the inverse association after the first 2 exposure days was more substantial in the late hot season. This suggests that physiological or behavioral acclimatization, although cannot be ruled out, is less likely to explain the attenuated association between heat exposure and hospitalization in Brazil over the course of the hot season. Alternatively, the lower cumulative risk associated with heat in the late hot season may be associated with medium-term harvesting: heat exposure in the early hot season may deplete the pool of susceptible individuals at the risk of hospitalization via triggering excess hospitalizations or deaths.

In comparison with all-cause studies, cause-specific investigations may shed further light on the association between heat exposure and health outcomes. In Brazil, exposure to early heat was associated with excess hospitalizations for a range of diseases, especially endocrine, nutritional, and metabolic diseases. This finding is in line with growing evidence that the impact of high temperature on the human body’s physiological systems may vary.^4,26^ The exception was for cardiovascular admissions, although the adverse effect of high temperature on the cardiovascular system has been well documented.^27^ In Brazil, we found that the risk of cardiovascular admissions associated with heat was minimal and negative in the early and late hot season. Several European studies have reported similar findings, explaining the absence of a positive association between heat exposure and cardiovascular admissions as a result of an excess number of sudden cardiac deaths occurring out of hospital.^28,29^ This explanation is consistent with the much lower risk of cardiovascular admissions in the early and late hot season during longer lag periods.

Our cause-specific analyses showed that only respiratory admissions were increasingly associated with heat exposure from the early to late hot season. Without information at the individual patient level, we were unable to provide a detailed explanation for this phenomenon. However, the different lag patterns in the early and late hot season indicate a speculation that is not unrealistic, which warrants further investigation: despite its harvesting effect in reducing vulnerable cases, exposure to early heat may have also increased survivors’ susceptibility in the late hot season.

Our study adds to the limited knowledge regarding the demographic and geographic characteristics of heat susceptibility over the course of the hot season. In Brazil, the association between heat exposure and hospitalization was strongest in young children, which may reflect their having an immature automatic thermoregulatory response relative to older children and adults (eg, greater heat absorption per unit of mass and immature ability to lose heat through sweating).^30,31^ The temporal increase in heat susceptibility for children is in agreement with previous findings that have reported children to have lower capacity of acclimatization compared with adults.^32^ The risk of hospitalization associated with heat exposure was also greater among individuals aged 80 years or older, possibly owing to age-associated physiological deterioration in body systems rendering them more susceptible to early heat.^26^ Similar to previous studies,^33^ we observed that among individuals aged 50 to 79 years there was an inverse association between heat exposure and risk of hospitalization in the late hot season. The lag patterns of the association between heat and hospitalization were largely consistent with admissions due to cardiovascular diseases, the leading health disorder for this age group.^34^ This may partly explain the inverse association between heat and hospitalization in the late hot season. Similarly, we observed the same lag patterns of association in children aged 5 to 9 years and in respiratory admissions, the leading cause of hospital admissions among young children.^34^ However, there may be other explainable factors that should not be overlooked, such as behavioral characteristics.

In the early hot season, the strength of the association between heat exposure and hospitalization increased from the hottest (the north) to more moderate areas (the northeast and central west) in Brazil. This is consistent with our previous finding that individuals in hot regions are less affected by high temperature relative to cooler areas.^35^ However, geographic adaptation cannot explain the weaker association between heat exposure and hospitalization in the coolest southeast and south regions. We speculate that the greatest level of economic development in southern Brazil may play a modifying role, leading to less susceptibility to high temperature for local populations.^26,36^

This study has several strengths. First, to our knowledge this is the first study to assess the nationwide temporal change in the association between heat exposure and hospitalization over the course of the hot season. The wide national coverage suggests that our findings are reliable and representative of the general situation in Brazil. Second, this is also the first study to explore the geographic, demographic, and cause-specific variations in the impact of the timing of heat exposure during the hot season. Our findings, particularly the age-specific results that used finer groupings than previous studies, provide evidence regarding the variation in response to heat exposure across the population over the hot season. Third, the climatic and geographic diversity of Brazil suggest our findings may also be applied to other South American countries.

### Limitations

We have to acknowledge several limitations. We applied grid temperature data owing to the lack of exposure information at the individual level. A previous study suggested that this may introduce measurement errors; however, if this is true then any error will be randomly distributed across the population resulting in an underestimation of the heat effect.^37^ In addition, we were unable to control for air pollution in the model because few Brazilian cities have data on air pollutants. However, numerous studies have indicated that the confounding effect of air pollutants on the association between temperature and health outcomes is minimal.^26,35^ We were unable to speculate on causal mechanisms mediating the risk of hospitalization associated with heat owing to the lack of information at the individual patient level. This is also a limitation for other ecological studies to investigate potentially complex causal mechanisms linking environmental exposures (eg, suboptimal ambient temperature and air pollution) and health outcomes.^38,39^

## Conclusions

In Brazil, the association between heat exposure and hospitalization was stronger in the early hot season, particularly for residents living in the central west and northeast regions. The risk of hospitalization associated with early heat exposure was not equally distributed across the Brazilian population. Children, elderly individuals, and patients admitted for endocrine, nutritional, and metabolic diseases were most susceptible to early heat exposure. The attenuated association suggests that people should take measures to protect their health from extreme high temperatures in the early hot season. The burden of heat-related morbidity is likely to continue to grow, and with it comes the need for better understanding of how human societies respond to, adapt to, and mitigate the effects of heat on health outcomes.

## Notes

Supplement. **eTable 1.** Causes of Hospitalizations and ICD-10 Codes **eTable 2.** Percentage Increase in the Risk of Hospitalization (With 95% Confidence Intervals) for Every 5°C Increase in Daily Mean Temperature During Early and Late Hot Season by Region **eTable 3.** Percentage Increase in the Risk of Hospitalization (With 95% Confidence Intervals) for Every 5°C Increase in Daily Mean Temperature During Early and Late Hot Season by Sex and Age **eTable 4.** Percentage Increase in the Risk of Hospitalization (With 95% Confidence Intervals) for Every 5°C Increase in Daily Mean Temperature During Early and Late Hot Season by Cause Category **eTable 5.** Results of Sensitivity Analyses: Percentage Increase in the Risk of Hospitalization for Every 5°C Increase in Daily Mean Temperature (With 95% Confidence Intervals) **eFigure 1.** Locations of 1,814 Brazilian Cities **eFigure 2.** Percentage Increase in the Risk of Hospitalization (With 95% Confidence Intervals) for Every 5°C Increase in Daily Mean Temperature Over Lag 0−7 Days During Early and Late Hot Season by Region **eFigure 3.** Percentage Increase in the Risk of Hospitalization (With 95% Confidence Intervals) for Every 5°C Increase in Daily Mean Temperature Over Lag 0−7 Days During Early and Late Hot Season by Sex and Age **eFigure 4.** Percentage Increase in the Risk of Hospitalization (With 95% Confidence Intervals) for Every 5°C Increase in Daily Mean Temperature Over Lag 0−7 Days During Early and Late Hot Season by Cause Category **eFigure 5.** Associations Between Daily Mean Temperature and Hospitalization During the Whole, Early and Late Hot Season Between 2000 and 2015 (at the National Level)
