# Supplementary material for: Assessment of Intraseasonal Variation in Hospitalization Associated With Heat Exposure in Brazil
Source: JAMA Netw Open. 2019 Feb 8;2(2):e187901. doi: 10.1001/jamanetworkopen.2018.7901 (PMC6484586; doi:10.1001/jamanetworkopen.2018.7901)
Supplement: Supplement. — eTable 1. Causes of Hospitalizations and ICD-10 Codes eTable 2. Percentage Increase in the Risk of Hospitalization (With 95% Confidence Intervals) for Every 5°C Increase in Daily Mean Temperature During Early and Late Hot Season by Region eTable 3. Percentage Increase in the Risk of Hospitalization (With 95% Confidence Intervals) for Every 5°C Increase in Daily Mean Temperature During Early and Late Hot Season by Sex and Age eTable 4. Percentage Increase in the Risk of Hospitalization (With 95% Confidence Intervals) for Every 5°C Increase in Daily Mean Temperature During Early and Late Hot Season by Cause Category eTable 5. Results of Sensitivity Analyses: Percentage Increase in the Risk of Hospitalization for Every 5°C Increase in Daily Mean Temperature (With 95% Confidence Intervals) eFigure 1. Locations of 1,814 Brazilian Cities eFigure 2. Percentage Increase in the Risk of Hospitalization (With 95% Confidence Intervals) for Every 5°C Increase in Daily Mean Temperature Over Lag 0−7 Days During Early and Late Hot Season by Region eFigure 3. Percentage Increase in the Risk of Hospitalization (With 95% Confidence Intervals) for Every 5°C Increase in Daily Mean Temperature Over Lag 0−7 Days During Early and Late Hot Season by Sex and Age eFigure 4. Percentage Increase in the Risk of Hospitalization (With 95% Confidence Intervals) for Every 5°C Increase in Daily Mean Temperature Over Lag 0−7 Days During Early and Late Hot Season by Cause Category eFigure 5. Associations Between Daily Mean Temperature and Hospitalization During the Whole, Early and Late Hot Season Between 2000 and 2015 (at the National Level) [file jamanetwopen-2-e187901-s001.pdf]

Supplementary Online Content

Zhao Q, Li S, Coelho MSZS, et al. Assessment of intraseasonal variation in hospitalization associated with heat exposure in Brazil. *JAMA Netw Open*. 2019;2(2):e187901. doi:10.1001/jamanetworkopen.2018.7901

eTable 1. Causes of Hospitalizations and ICD-10 Codes

eTable 2. Percentage Increase in the Risk of Hospitalization (With 95% Confidence Intervals) for Every 5°C Increase in Daily Mean Temperature During Early and Late Hot Season by Region

eTable 3. Percentage Increase in the Risk of Hospitalization (With 95% Confidence Intervals) for Every 5°C Increase in Daily Mean Temperature During Early and Late Hot Season by Sex and Age

eTable 4. Percentage Increase in the Risk of Hospitalization (With 95% Confidence Intervals) for Every 5°C Increase in Daily Mean Temperature During Early and Late Hot Season by Cause Category

eTable 5. Results of Sensitivity Analyses: Percentage Increase in the Risk of Hospitalization for Every 5°C Increase in Daily Mean Temperature (With 95% Confidence Intervals)

eFigure 1. Locations of 1,814 Brazilian Cities

eFigure 2. Percentage Increase in the Risk of Hospitalization (With 95% Confidence Intervals) for Every 5°C Increase in Daily Mean Temperature Over Lag 0−7 Days During Early and Late Hot Season by Region

eFigure 3. Percentage Increase in the Risk of Hospitalization (With 95% Confidence Intervals) for Every 5°C Increase in Daily Mean Temperature Over Lag 0−7 Days During Early and Late Hot Season by Sex and Age

eFigure 4. Percentage Increase in the Risk of Hospitalization (With 95% Confidence Intervals) for Every 5°C Increase in Daily Mean Temperature Over Lag 0−7 Days During Early and Late Hot Season by Cause Category

eFigure 5. Associations Between Daily Mean Temperature and Hospitalization During the Whole, Early and Late Hot Season Between 2000 and 2015 (at the National Level)

This supplementary material has been provided by the authors to give readers additional information about their work.

© 2019 Zhao Q et al. *JAMA Network Open.*

eTable 1. Causes of hospitalizations and ICD-10 codes.

No.	Causes	ICD-10 codes
1	Endocrine, nutritional and metabolic diseases	E00-E90
2	Diseases of the cardiovascular system	I00-I99
3	Diseases of the respiratory system	J00-J99
4	Diseases of the genitourinary system	N00-N99
5	Maternal conditions	O00-O99
6	Certain conditions originating in the perinatal period	P00-P99
7	Injury, poisoning and certain other consequences of external causes	S00-T98

© 2019 Zhao Q et al. *JAMA Network Open.*

eTable 2. Percentage increase in the risk of hospitalization (with 95% confidence intervals) for every 5°C increase in daily mean temperature during early and late hot season by region. Regional difference during early or late hot season, and intraseasonal difference for each region were examined.

	Early hot season				Late hot season				Intraseasonal difference	
	**Increase in hospitalization (%)**	**I^2 value**	***P* value [a]**		**Increase in hospitalization (%)**	**I^2 value**	***P* value [b]**		**Difference (%)**	***P* value [c]**
**National**	4.6 (4.3, 4.9)	32.29	–		2.3 (1.9, 2.6)	32.55	–		2.3 (1.8, 2.7)	< .001
**Region**										
North	3.8 (0.9, 6.7)	23.38	.80		-0.4 (-2.9, 2.2)	18.45	.35		4.4 (0.5, 8.5)	.03
Northeast	6.4 (5.5, 7.3)	24.27	< .001		3.9 (3.0, 4.7)	22.03	< .001		2.4 (1.2, 3.6)	< .001
Central West	7.1 (6.1, 8.2)	22.38	< .001		2.1 (1.1, 3.0)	20.89	.03		4.9 (3.5, 6.4)	< .001
Southeast	4.1 (3.7, 4.5)	18.24	.07		2.7 (2.3, 3.1)	19.84	< .001		1.4 (0.8, 2.0)	< .001
South	3.6 (3.0, 4.2)	48.16	Reference		0.8 (0.2, 1.6)	53.06	Reference		2.8 (1.9, 3.8)	< .001

Note: [a,b] P values of significant tests examining regional difference in the risk of hospitalization associated with heat exposure during early or late hot season, respectively, with the south set as the reference. [c] P values of significant tests examining intraseasonal difference in the risk of hospitalization associated with heat exposure for each region, with the late hot season set as the reference.

© 2019 Zhao Q et al. *JAMA Network Open.*

eTable 3. Percentage increase in the risk of hospitalization (with 95% confidence intervals) for every 5°C increase in daily mean temperature during early and late hot season by sex and age. Subgroup difference during early or late hot season, and intraseasonal difference for each population subgroup were examined

	Early hot season				Late hot season				Intraseasonal difference	
	**Increase in hospitalization (%)**	**I^2 value**	***P* value [a]**		**Increase in hospitalization (%)**	**I^2 value**	***P* value [b]**		**Difference (%)**	***P* value [c]**
**Sex**										
Men	4.8 (4.4, 5.3)	21.25	0.08		1.9 (1.4, 2.3)	19.13	0.05		2.9 (2.3, 3.5)	< .001
Women	4.3 (4.0, 4.7)	25.56	Reference		2.5 (2.1, 2.9)	25.73	Reference		1.8 (1.3, 2.3)	< .001
**Age (years)**										
0−4	10.2 (9.2, 11.1)	31.20	< .001		12.3 (11.4, 13.3)	25.34	< .001		-2.0 (-3.2, -0.8)	< .001
5−9	8.1 (6.8, 9.3)	5.27	< .001		7.1 (5.8, 8.5)	4.85	< .001		0.9 (-0.8, 2.6)	0.30
10−19	5.5 (4.9, 6.1)	3.35	< .001		4.3 (3.7, 4.9)	2.86	< .001		1.1 (0.3, 2.0)	0.008
20−29	4.6 (4.1, 5.2)	4.65	< .001		2.9 (2.3, 3.4)	3.92	< .001		1.7 (1.0, 2.4)	< .001
30−39	4.4 (3.8, 5.0)	6.92	< .001		1.7 (1.0, 2.3)	7.51	< .001		2.7 (1.8, 3.6)	< .001
40−49	3.2 (2.4, 4.0)	15.57	0.02		-0.6 (-1.4, 0.2)	14.70	0.03		3.8 (2.7, 4.9)	< .001
50−59	2.2 (1.5, 2.9)	5.65	0.60		-1.3 (-2.1, -0.6)	6.96	0.32		3.6 (2.5, 4.6)	< .001
60−69	1.9 (1.1, 2.6)	6.90	Reference		-1.9 (-2.6, -1.1)	8.12	Reference		3.8 (2.7, 5.0)	< .001
70−79	2.2 (1.4, 3.0)	11.11	0.59		-1.7 (-2.5, -0.9)	11.84	0.75		4.0 (2.8, 5.2)	< .001
≥ 80	5.7 (4.7, 6.7)	8.36	< .001		1.9 (0.9, 2.9)	6.23	< .001		3.8 (2.4, 5.2)	< .001

Note: [a,b] *P* values of significant tests examining sex and age differences in the risk of hospitalization associated with heat exposure during early or late hot season. Women and population subgroups aged 60−69 years were set as the references. [c] *P* values of significant tests examining intraseasonal difference in the risk of hospitalization associated with heat exposure for each population subgroup, with the late hot season set as the reference.

© 2019 Zhao Q et al. *JAMA Network Open.*

eTable 4. Percentage increase in the risk of hospitalization (with 95% confidence intervals) for every 5°C increase in daily mean temperature during early and late hot season by cause category. Cause-specific difference during early or late hot season, and intraseasonal difference for each cause were examined

	**Early hot season**				**Late hot season**				**Intraseasonal difference**	
	**Increase in hospitalization (%)**	**I^2 value**	***P* value [a]**		**Increase in hospitalization (%)**	**I^2 value**	***P* value [b]**		**Difference (%)**	***P* value [c]**
Endocrine, nutritional, metabolic diseases	12.3 (10.9, 13.7)	6.46	< .001		7.6 (6.2, 9.0)	4.53	< .001		4.3 (2.5, 6.2)	< .001
Perinatal conditions	8.3 (6.7, 9.9)	8.94	< .001		7.7 (6.0, 9.4)	5.95	< .001		0.5 (-1.7, 2.7)	0.65
Injury, poisoning	6.6 (5.8, 7.5)	9.12	< .001		4.4 (3.5, 5.3)	8.32	< .001		2.2 (1.0, 3.3)	< .001
Genitourinary diseases	6.5 (5.6, 7.4)	12.66	< .001		3.8 (2.8, 4.7)	10.04	< .001		2.6 (1.3, 3.8)	< .001
Respiratory diseases	4.1 (3.3, 4.9)	30.24	< .001		6 (5.1, 6.9)	30.47	< .001		-1.7 (-2.8, -0.6)	0.002
Maternal conditions	2.7 (2.3, 3.1)	5.82	< .001		2.1 (1.6, 2.6)	4.35	< .001		0.6 (-0.1, 1.2)	0.08
Cardiovascular diseases	-0.6 (-1.2, 0.1)	12.48	< .001		-3.9 (-4.6, -3.3)	10.57	< .001		3.5 (2.5, 4.4)	< .001

Note: [a,b] *P* values of significant tests examining cause-specifc differences in the risk of hospitalization associated with heat exposure during early or late hot season. Hospitalizations for cardiovascular diseases were set as the references. [c] *P* values of significant tests examining intraseasonal difference in the risk of hospitalization associated with heat exposure for each cause subgroup, with the late hot season set as the reference.

© 2019 Zhao Q et al. *JAMA Network Open.*

eTable 5. Results of sensitivity analyses: percentage increase in the risk of hospitalization for every 5°C increase in daily mean temperature (with 95% confidence intervals). Data analyses were performed by changing maximum lag for daily mean temperature from 7 to 9 days and the degrees of freedom (df) from 3 to 6 (with data of 1,814 cities), and adjusting for relative humidity (with observed data of 193 cities).

Group	Percentage increase in risk of hospitalization, %	
	Early hot season	Late hot season
**1,814 cities**		
Primary model	4.6 (4.3, 4.9)	2.3 (1.9, 2.6)
Lag 0−8 days	4.2 (3.9, 4.6)	1.9 (1.5, 2.2)
Lag 0−9 days	4.0 (3.6, 4.3)	1.6 (1.3, 2.0)
Df = 4	4.5 (4.2, 4.8)	2.2 (1.8, 2.5)
Df = 5	4.8 (4.4, 5.1)	2.5 (2.1, 2.8)
Df = 6	4.8 (4.5, 5.1)	2.5 (2.1, 2.8)
**193 cities**		
Primary model	5.3 (4.3, 6.2)	2.0 (1.1, 3.0)
Adjustment for relative humidity	4.8 (3.8, 5.9)	1.7 (0.7, 2.7)

© 2019 Zhao Q et al. *JAMA Network Open.*

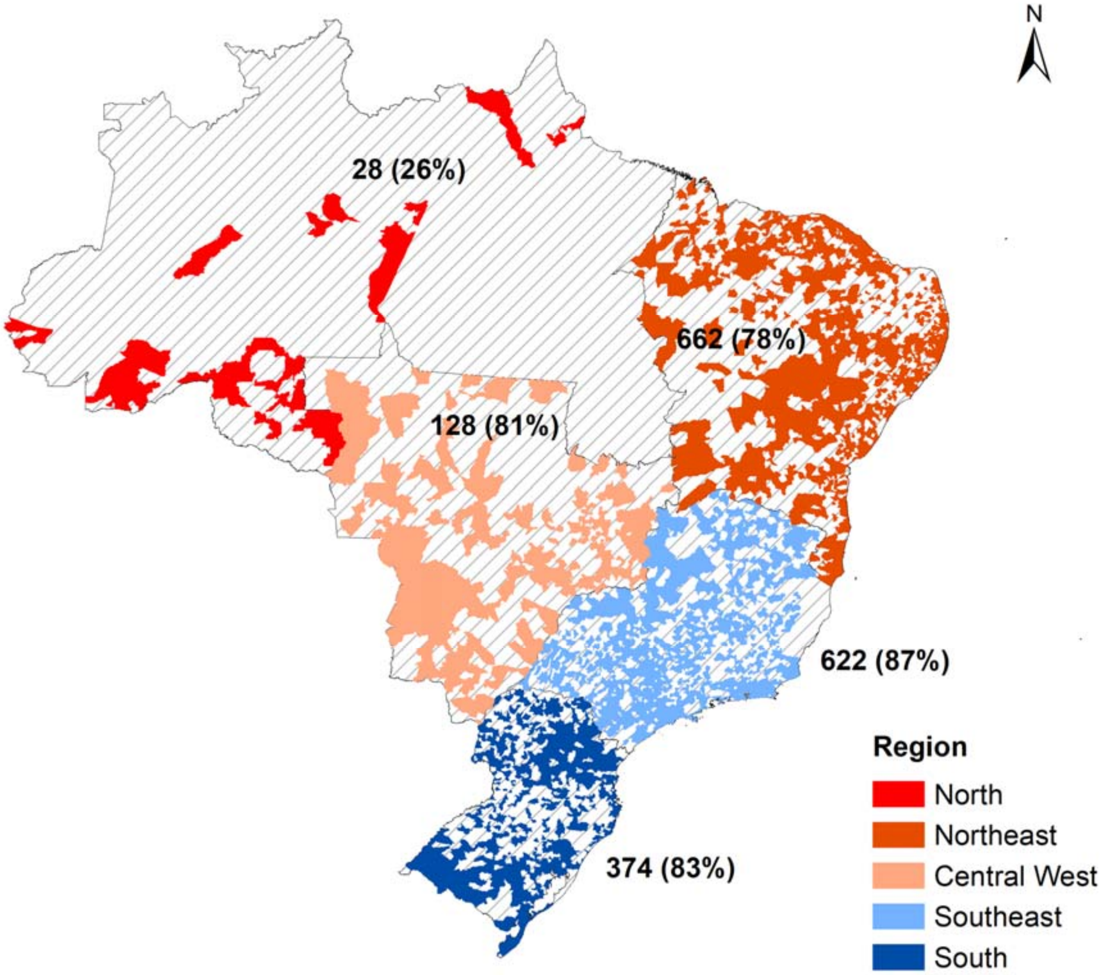

eFigure 1. Locations of 1,814 Brazilian cities.
The number of cities in each region with population coverage (%) are provided.

© 2019 Zhao Q et al. *JAMA Network Open.*

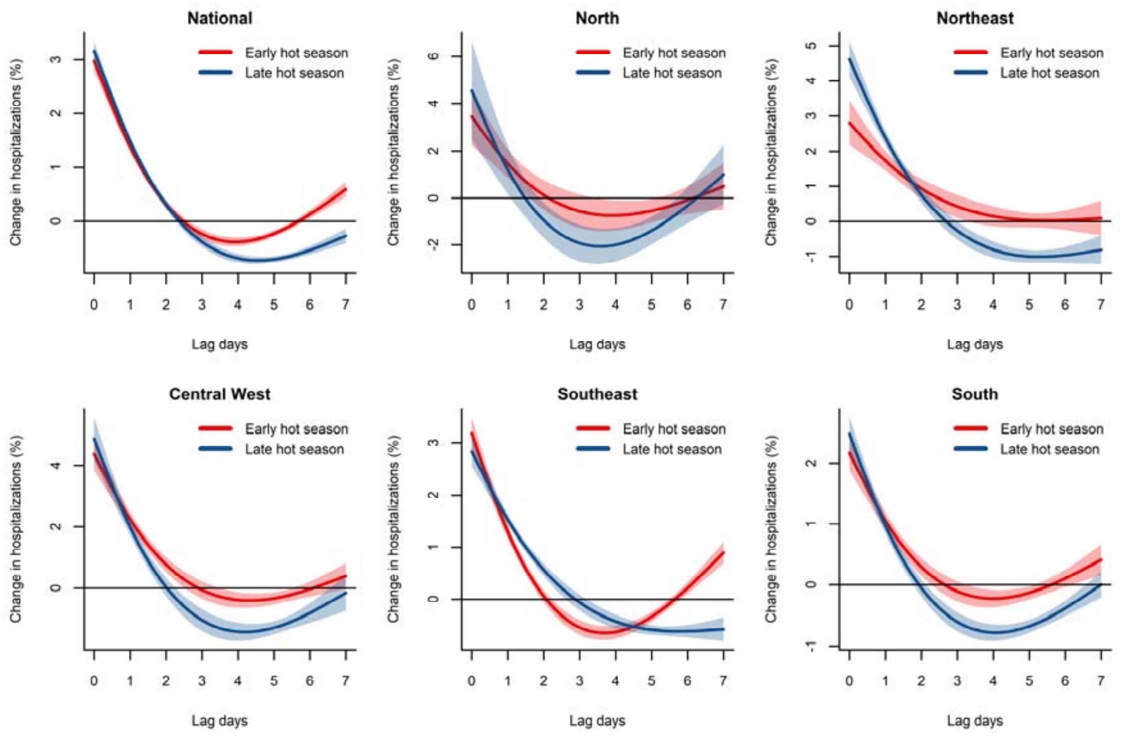

eFigure 2. Percentage increase in the risk of hospitalization (with 95% confidence intervals) for every 5°C increase in daily mean temperature over lag 0−7 days during early and late hot season by region.

© 2019 Zhao Q et al. *JAMA Network Open.*

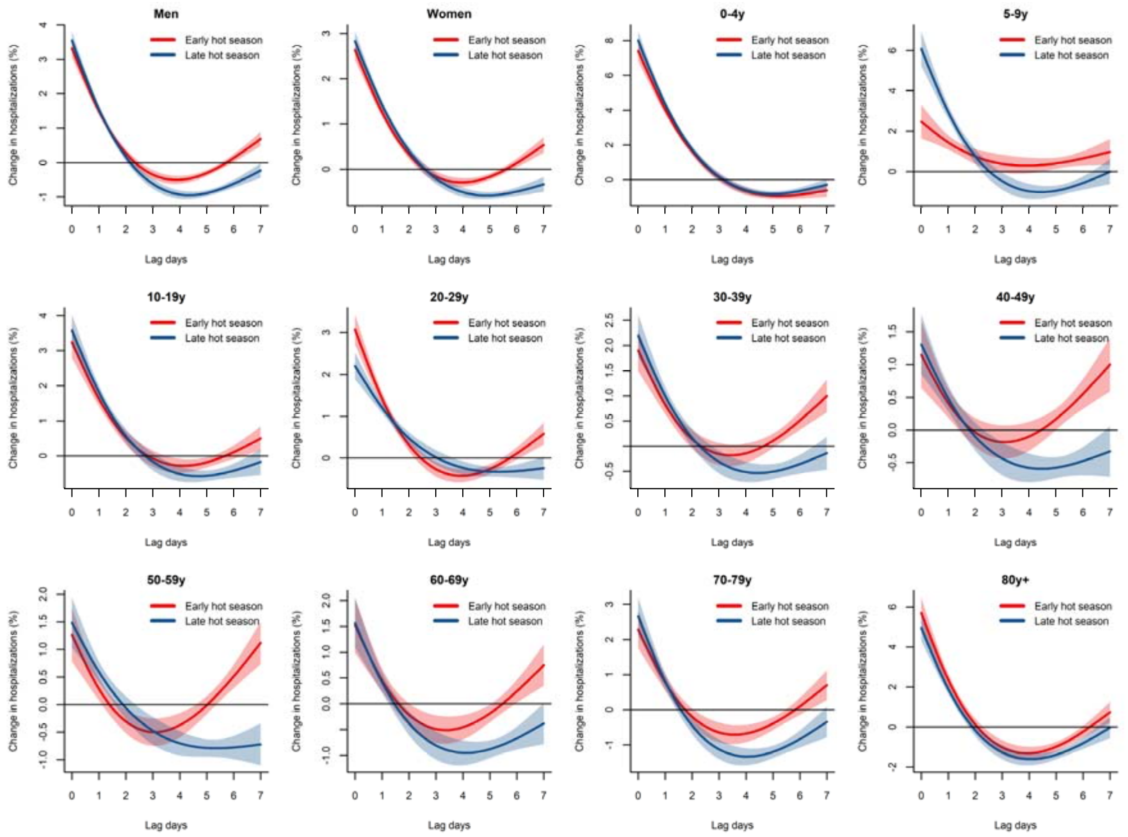

eFigure 3. Percentage increase in the risk of hospitalization (with 95% confidence intervals) for every 5°C increase in daily mean temperature over lag 0−7 days during early and late hot season by sex and age.

© 2019 Zhao Q et al. *JAMA Network Open.*

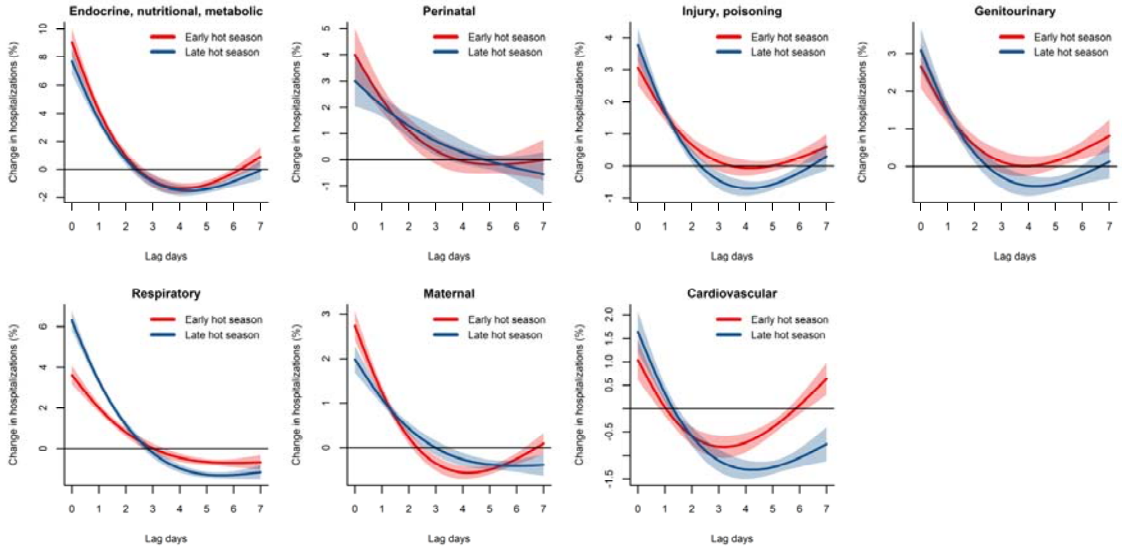

eFigure 4. Percentage increase in the risk of hospitalization (with 95% confidence intervals) for every 5°C increase in daily mean temperature over lag 0−7 days during early and late hot season by cause category.

© 2019 Zhao Q et al. *JAMA Network Open.*

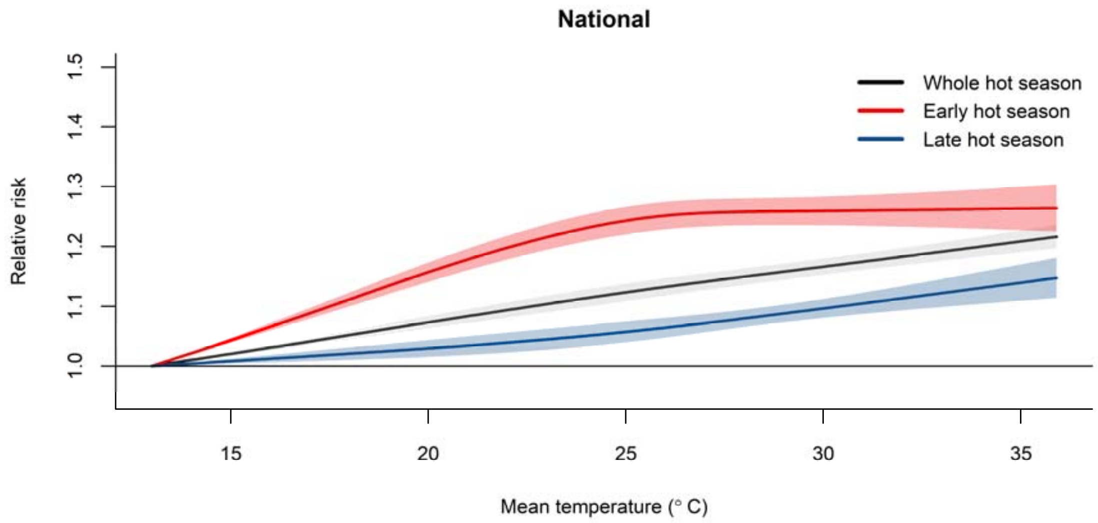

eFigure 5. Associations between daily mean temperature and hospitalization during the whole, early and late hot season between 2000 and 2015 (at the national level). A distributed lag non-linear model was applied, with a natural cubic spline with three degrees of freedom for daily temperature.

© 2019 Zhao Q et al. *JAMA Network Open.*
